# Association of indocyanine green fluorescence navigation with extrahepatic biliary anatomy identification and operative efficiency in laparoscopic cholecystectomy: a retrospective cohort study

**DOI:** 10.3389/fmed.2026.1869510

**Published:** 2026-08-07

**Authors:** Jingyi Xu, Lei Yang, Shuang Wang, Liusheng Wu, Yuehua Liang, Xialin Xie, Wenqiang Wang, Lu Gao, Jun Yan

**Affiliations:** 1 Department of Liver Surgery, Beijing Tsinghua Changgung Hospital, School of Clinical Medicine, Tsinghua Medicine, Tsinghua University, Beijing, China; 2 Department of Basic Medicine, Beijing Health Vocational College, Beijing, China

**Keywords:** critical view of safety, extrahepatic biliary anatomy, fluorescence, cholangiography, indocyanine green, laparoscopic cholecystectomy

## Abstract

**Background:**

Indocyanine green (ICG) fluorescence cholangiography may improve visualization of the extrahepatic biliary tree during laparoscopic cholecystectomy (LC). We examined whether its use was associated with biliary structure identification and with two time-based measures of operative efficiency in patients undergoing LC for benign gallbladder disease.

**Methods:**

Consecutive adult patients assessed for cholecystectomy for benign gallbladder disease between January and December 2025 were retrospectively screened. After the ICG-assisted and conventional white-light approaches had been explained, patients chose their preferred procedure; allocation was therefore non-random. The primary outcome was complete identification of the cystic duct, common bile duct, common hepatic duct, and cystic duct-common bile duct junction. The operating surgeon recorded identification during surgery, and a senior physician subsequently reviewed the operative video. Operative efficiency comprised time to achieve the critical view of safety (CVS) and total operative time. Drain placement was analyzed separately. Multivariable models were used to adjust for clinically relevant covariates.

**Results:**

Among 198 patients, 103 underwent ICG fluorescence-assisted LC and 95 underwent conventional white-light LC. All four biliary structures were identified in 91.3% of the ICG group and 56.8% of the white-light group (*p* < 0.001). Median time to CVS was 12.0 versus 21.0 min (*p* < 0.001), median operative time was 36.0 versus 48.0 min (*p* < 0.001), and drains were placed in 17.5% versus 38.9% of patients (*p* < 0.001). The associations remained significant after multivariable adjustment. No BDI, bile leakage, conversion to open surgery, reoperation, or ICG-related adverse event occurred.

**Conclusion:**

ICG fluorescence use was associated with more frequent identification of extrahepatic biliary structures and shorter time to CVS and completion of LC. Because patients selected the surgical approach and assessment of anatomical identification included clinical judgment, these results should be interpreted as observational. They do not show that ICG reduces the risk of BDI. Prospective multicenter studies are needed.

## 1 Introduction

Laparoscopic cholecystectomy (LC) is the standard surgical treatment for symptomatic benign gallbladder diseases, including gallstones, acute cholecystitis, chronic cholecystitis, gallbladder polyps, and gallbladder adenomyomatosis (1–3). Although LC is widely performed and generally safe, bile duct injury (BDI) remains one of the most serious complications of this procedure (1, 2). BDI may lead to bile leakage, biliary stricture, recurrent cholangitis, repeated endoscopic or surgical interventions, impaired long-term quality of life, increased healthcare costs, and even mortality (2, 4). Therefore, improving intraoperative recognition of extrahepatic biliary anatomy remains a central component of safe laparoscopic biliary surgery.

Misidentification of biliary anatomy is a major mechanism underlying BDI during LC (1, 2). This risk may be increased in patients with acute inflammation, chronic fibrosis, dense adhesions, gallbladder wall thickening, contracted gallbladder, obesity, or anatomical variation of the biliary tree (3, 5–7). The critical view of safety (CVS) has therefore been recommended as a key intraoperative strategy before clipping and dividing the cystic duct and cystic artery (1, 4, 5). However, achieving CVS may be technically difficult in complex gallbladder cases, and conventional white-light imaging does not always provide sufficient visual information for early and reliable recognition of the cystic duct, common bile duct, common hepatic duct, and cystic duct-common bile duct junction (6, 7).

Current safe cholecystectomy strategies emphasize standardized dissection, correct interpretation of CVS, timely use of bailout procedures, and adjunctive imaging when biliary anatomy is unclear (1, 2, 6, 7). Intraoperative cholangiography (IOC) can delineate the biliary tree and identify common bile duct stones, but its routine use remains debated because it requires cystic duct cannulation, additional operative steps, radiation exposure, specific equipment, and technical expertise (8, 9). These limitations have encouraged the development of real-time, non-radiative, and less invasive imaging modalities for intraoperative biliary navigation.

Near-infrared (NIR) fluorescence imaging with indocyanine green (ICG) has emerged as a practical adjunct for biliary visualization during LC (10–16). After intravenous administration, ICG binds to plasma proteins, is taken up by hepatocytes, and is excreted into bile, allowing bile-containing structures to be visualized under NIR fluorescence imaging (10, 11). Compared with IOC, ICG fluorescence navigation does not require cystic duct cannulation or radiation exposure and can be repeatedly applied during different stages of dissection. By switching between white-light and fluorescence modes, surgeons can obtain additional anatomical information while maintaining the standard laparoscopic workflow.

Recent randomized controlled trials, cohort studies, and systematic reviews have increasingly evaluated the clinical value of ICG fluorescence cholangiography in LC (12–19). The FALCON international multicenter randomized controlled trial showed that NIR fluorescence cholangiography allowed earlier identification of relevant extrahepatic biliary anatomy during LC (12). Another randomized controlled trial in emergency LC suggested that routine ICG use may enhance operative safety in acute gallbladder disease (13). A 2025 systematic review and meta-analysis with trial sequential analysis, restricted to randomized controlled trials, reported that ICG fluorescence cholangiography significantly improved the odds of successful common bile duct identification, although its effects on rare patient-centered outcomes such as BDI, bile leakage, conversion to open surgery, and reoperation remain uncertain (17). Other recent meta-analyses and comparative studies have similarly suggested that ICG fluorescence imaging may improve biliary visualization and surgical efficiency without increasing perioperative risk (18–21).

Evidence from recent trials is encouraging, but the performance of ICG fluorescence in routine LC for benign gallbladder disease is not fully established. We therefore compared ICG fluorescence-assisted LC with conventional white-light LC in a retrospective cohort. The primary outcome was complete identification of the cystic duct, common bile duct, common hepatic duct, and cystic duct-common bile duct junction. Time to CVS and total operative time were used as the two measures of operative efficiency. Other outcomes included blood loss, conversion to open surgery, drain use and duration, postoperative length of stay, complications, 30-day readmission, and ICG-related adverse events. We expected ICG use to be associated with better anatomical identification and shorter operative times without an increase in perioperative adverse events.

## 2 Methods

### 2.1 Study design and patients

This retrospective, single-center cohort study was conducted at Beijing Tsinghua Changgung Hospital. Consecutive adult patients assessed for cholecystectomy for benign gallbladder disease between January and December 2025 were retrospectively screened. The study protocol was reviewed and approved by the Ethics Committee of Beijing Tsinghua Changgung Hospital (approval no. 26463-4-01). Written informed consent was obtained from all participants. The study was conducted in accordance with the Declaration of Helsinki and applicable local regulations.

Before surgery, all patients received an explanation of conventional white-light LC and ICG fluorescence-assisted LC, including the principal features, potential benefits, and limitations of each approach. Treatment allocation was not randomized. After counseling, each patient independently selected the preferred surgical approach; patient preference was therefore the determinant of ICG use and group assignment. Patients selecting ICG fluorescence-assisted LC comprised the ICG fluorescence group, whereas those selecting conventional white-light LC comprised the conventional white-light group.

The inclusion criteria were as follows: patients aged 18 years or older; patients diagnosed with benign gallbladder diseases, including gallstones, acute cholecystitis, chronic cholecystitis, gallbladder polyps, or gallbladder adenomyomatosis; patients who underwent laparoscopic cholecystectomy; and patients with complete perioperative clinical data.

The exclusion criteria were suspected or confirmed gallbladder malignancy or biliary malignancy, preoperative planned open cholecystectomy, known allergy to ICG or iodine-containing agents, pregnancy or lactation, severe organ dysfunction contraindicating laparoscopic surgery, liver cirrhosis, human immunodeficiency virus infection, and incomplete clinical records.

During the study period, 209 patients were assessed for eligibility. Eleven patients were excluded, including 2 with suspected or confirmed gallbladder or biliary malignancy, 5 with contraindications to laparoscopic surgery for various reasons, 2 with a preoperative plan for open cholecystectomy, and 2 without available intraoperative video recordings. The remaining 198 patients were included in the final analysis, including 103 in the ICG fluorescence group and 95 in the conventional white-light group.

**Figure 1:**
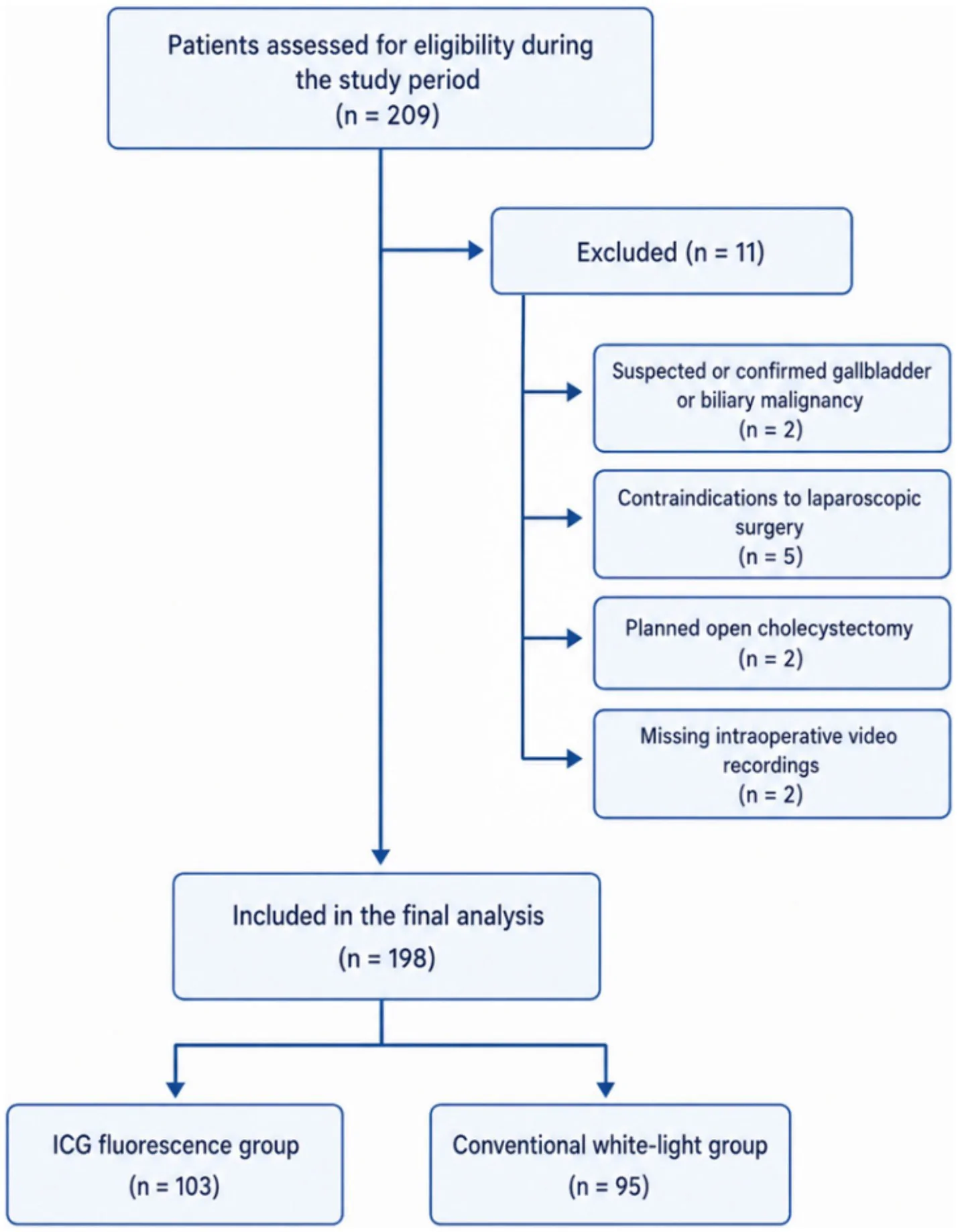
Patient selection flowchart. Flowchart showing patient screening, reasons for exclusion, and final group allocation.

A patient selection flowchart is presented in Figure 1.

### 2.2 Surgical procedure

All procedures were performed under general anesthesia by surgeons experienced in laparoscopic biliary surgery. A standard laparoscopic cholecystectomy was performed using a conventional multi-port technique.

In the ICG fluorescence group, ICG was supplied as a 25-mg vial (Dandong Yichuang Pharmaceutical Co., Ltd., Dandong, China; National Medical Products Administration approval no. H20055881). Each vial was reconstituted with 10 mL of sterile water for injection, yielding a final concentration of 2.5 mg/mL. A 0.4-mL aliquot, corresponding to a fixed dose of 1 mg ICG, was administered through a peripheral intravenous line 30 min before surgery.

Near-infrared fluorescence imaging was performed using one of the following fluorescence laparoscopic systems: OPTO-CAM214K/FloNavi 214 K (Guangdong OptoMedic Technologies, Inc., Guangdong, China), SV-M4K40 (SonoScape Medical Corp., Shenzhen, China), or DPM-END0CAM-02 (Beijing Digital Precision Medicine Technology Co., Ltd., Beijing, China). Fluorescence mode was activated at the beginning of the operation and remained available throughout the procedure. The surgeon alternated between conventional white-light and fluorescence modes as required to assess the extrahepatic biliary anatomy.

During surgery, the gallbladder was retracted to expose Calot’s triangle. The cystic duct and cystic artery were carefully dissected, and CVS was achieved before clipping and division whenever technically feasible. In the conventional white-light group, LC was performed using standard white-light imaging without fluorescence navigation. Conventional radiographic intraoperative cholangiography was not used in either group. The use of subtotal cholecystectomy and other bailout or damage-avoidance procedures was recorded.

Drainage placement was determined by the operating surgeon according to intraoperative findings, including local inflammation, oozing, bile contamination, difficult dissection, or concern for postoperative fluid collection.

### 2.3 Data collection

Baseline demographic and clinical characteristics were collected, including age, sex, body mass index, American Society of Anesthesiologists classification, hypertension, diabetes mellitus, previous abdominal surgery, disease type, gallbladder wall thickness, preoperative white blood cell count, C-reactive protein, alanine aminotransferase, and aspartate aminotransferase.

Intraoperative variables included identification of extrahepatic biliary structures, time to achieve the CVS, operative time, intraoperative blood loss, conversion to open surgery, drainage placement, and drainage duration.

Postoperative outcomes included postoperative hospital stay, BDI, bile leakage, intra-abdominal infection, incision infection, reoperation, 30-day readmission, and ICG-related adverse events.

### 2.4 Outcome definitions

The primary outcome was complete identification of all four predefined extrahepatic biliary structures: the cystic duct, common bile duct, common hepatic duct, and cystic duct-common bile duct junction. Individual identification rates for each structure were also analyzed.

Identification of each biliary structure was assessed in real time by the operating surgeon and documented in the operative record. After surgery, the operative video was reviewed by a senior physician to verify the recorded identification status. The postoperative video assessment served as the final endpoint determination. Any disagreement between the operating surgeon and the video reviewer was resolved by consensus. For the purposes of this analysis, a structure was considered identified when it was sufficiently visualized to be distinguished from adjacent tissues and its anatomical course could be followed within the operative field. The postoperative review was not conducted using a blinded, multiple-independent-reviewer adjudication protocol, and inter-rater agreement was not formally assessed.

Secondary outcomes included time to achieve CVS, operative time, intraoperative blood loss, conversion to open surgery, drainage placement, drainage duration, postoperative hospital stay, and postoperative complications. For this study, operative efficiency was operationally defined using the two time-based outcomes of time to achieve CVS and total operative time. Drain placement and drainage duration were analyzed separately as perioperative management outcomes and were not included in the definition of operative efficiency.

Time to achieve CVS was measured from the operative video and defined as the interval between the video timestamp marking the beginning of Calot’s triangle dissection and the timestamp at which CVS was fully exposed. Operative time was defined as the interval from the start of laparoscopic surgery to completion of the procedure according to the operative record. BDI was defined and classified according to the Strasberg classification (types A-E), encompassing leakage from the cystic duct stump or minor ducts, injuries involving aberrant right hepatic ducts, lateral injuries to major bile ducts, and major duct transection or stricture (2). Postoperative bile leakage was additionally recorded as a separate clinical complication on the basis of bilious drainage, clinical manifestations, imaging findings, or the need for intervention. A bile leak attributable to a Strasberg type A, C, or D injury would therefore have been reported under both categories.

### 2.5 Statistical analysis

Continuous variables were assessed for normality using the Shapiro–Wilk test. Normally distributed continuous variables were expressed as mean ± standard deviation and compared using Welch’s *t*-test. Non-normally distributed continuous variables were expressed as median and interquartile range and compared using the Mann–Whitney U test.

Categorical variables were expressed as number and percentage and compared using the chi-square test or Fisher’s exact test, as appropriate.

Multivariable analyses were performed to evaluate the association between ICG fluorescence navigation and clinically relevant outcomes. Continuous outcomes, including time to achieve the CVS, operative time, and postoperative hospital stay, were analyzed using multivariable linear regression with robust standard errors. Binary outcomes, including complete identification of all four biliary structures and drainage placement, were analyzed using multivariable logistic regression.

The adjusted covariates included age, sex, body mass index, American Society of Anesthesiologists classification, hypertension, diabetes mellitus, previous abdominal surgery, acute cholecystitis, and gallbladder wall thickness. Effect estimates were reported as regression coefficients or odds ratios with 95% confidence intervals. A two-sided *p* value <0.05 was considered statistically significant.

## 3 Results

### 3.1 Patient characteristics

During the study period, 209 patients were assessed for eligibility. Eleven patients were excluded: 2 because of suspected or confirmed gallbladder or biliary malignancy, 5 because of contraindications to laparoscopic surgery, 2 because open cholecystectomy was planned preoperatively, and 2 because intraoperative video recordings were unavailable (Figure 1).

The remaining 198 patients were included in the final analysis, including 103 patients in the ICG fluorescence group and 95 patients in the conventional white-light group.

The baseline characteristics of the two groups are summarized in Table 1. The median age was 51.0 years in the ICG fluorescence group and 53.0 years in the conventional white-light group, with no significant difference between groups. The proportion of male patients was also comparable between the two groups. Body mass index, gallbladder wall thickness, preoperative white blood cell count, C-reactive protein, alanine aminotransferase, aspartate aminotransferase, American Society of Anesthesiologists classification, diabetes mellitus, previous abdominal surgery, and disease type did not differ significantly between groups.

**Table 1:** Baseline characteristics of the two groups.

Variable	ICG fluorescence group (*n* = 103)	Conventional white-light group (*n* = 95)	Statistical test	*p* value
Age (years)	51.0 (37.0–67.5)	53.0 (36.5–66.5)	Mann–Whitney U test	0.885
BMI (kg/m^2^)	24.5 ± 2.8	24.3 ± 3.3	Welch *t*-test	0.582
Gallbladder wall thickness (mm)	4.1 (3.5–5.1)	4.5 (3.2–5.2)	Mann–Whitney U test	0.637
Preoperative WBC (×10^9^/L)	6.9 (4.8–8.7)	7.6 (5.4–8.9)	Mann–Whitney U test	0.139
Preoperative CRP (mg/L)	9.5 (4.0–15.0)	9.5 (5.3–15.1)	Mann–Whitney U test	0.634
Preoperative ALT (U/L)	32.3 ± 13.5	34.5 ± 15.1	Welch *t*-test	0.285
Preoperative AST (U/L)	26.4 ± 10.0	28.1 ± 12.6	Welch *t*-test	0.292
Male sex	49 (47.6)	43 (45.3)	Chi-square test	0.745
ASA classification			Chi-square test	0.847
ASA 1	35 (34.0)	36 (37.9)		
ASA 2	59 (57.3)	51 (53.7)		
ASA 3	9 (8.7)	8 (8.4)		
Hypertension	40 (38.8)	24 (25.3)	Chi-square test	0.041
Diabetes mellitus	18 (17.5)	14 (14.7)	Chi-square test	0.601
Previous abdominal surgery	13 (12.6)	17 (17.9)	Chi-square test	0.301
Disease type			Chi-square test	0.104
Gallstones	63 (61.2)	64 (67.4)		
Chronic cholecystitis	16 (15.5)	11 (11.6)		
Acute cholecystitis	16 (15.5)	10 (10.5)		
Gallbladder polyps	3 (2.9)	9 (9.5)		
Gallbladder adenomyomatosis	5 (4.9)	1 (1.1)		

Continuous variables are presented as mean ± SD or median (IQR) according to distribution; categorical variables are presented as *n* (%). BMI, body mass index; ALT, alanine aminotransferase; AST, aspartate aminotransferase.

However, the proportion of patients with hypertension was higher in the ICG fluorescence group than in the conventional white-light group, 40 of 103 patients, 38.8%, versus 24 of 95 patients, 25.3%, *p* = 0.041. This baseline imbalance was adjusted for in the subsequent multivariable analyses.

### 3.2 Extrahepatic biliary structure identification

The identification of extrahepatic biliary structures is shown in Table 2. The ICG fluorescence group had significantly higher identification rates of all predefined biliary structures compared with the conventional white-light group.

**Table 2:** Comparison of extrahepatic biliary structure identification between groups.

Variable	ICG fluorescence group (*n* = 103)	Conventional white-light group (*n* = 95)	Statistical test	*p* value
Cystic duct identification	103 (100.0)	84 (88.4)	Chi-square test	<0.001
Common bile duct identification	103 (100.0)	82 (86.3)	Chi-square test	<0.001
Common hepatic duct identification	97 (94.2)	78 (82.1)	Chi-square test	0.008
Cystic duct-common bile duct junction identification	100 (97.1)	83 (87.4)	Chi-square test	0.010
Complete identification of all four biliary structures	94 (91.3)	54 (56.8)	Chi-square test	<0.001

Data are presented as *n* (%). The four biliary structures include the cystic duct, common bile duct, common hepatic duct, and cystic duct-common bile duct junction.

The cystic duct was identified in 103 of 103 patients, 100.0%, in the ICG fluorescence group and in 84 of 95 patients, 88.4%, in the conventional white-light group, *p* < 0.001. The common bile duct was identified in 103 of 103 patients, 100.0%, in the ICG fluorescence group and in 82 of 95 patients, 86.3%, in the conventional white-light group, *p* < 0.001.

The common hepatic duct identification rate was also higher in the ICG fluorescence group than in the conventional white-light group, 97 of 103 patients, 94.2%, versus 78 of 95 patients, 82.1%, *p* = 0.008. Similarly, the cystic duct-common bile duct junction was identified more frequently in the ICG fluorescence group, 100 of 103 patients, 97.1%, than in the conventional white-light group, 83 of 95 patients, 87.4%, *p* = 0.010.

Complete identification of all four biliary structures was achieved in 94 of 103 patients, 91.3%, in the ICG fluorescence group, compared with 54 of 95 patients, 56.8%, in the conventional white-light group, *p* < 0.001.

**Figure 2:**
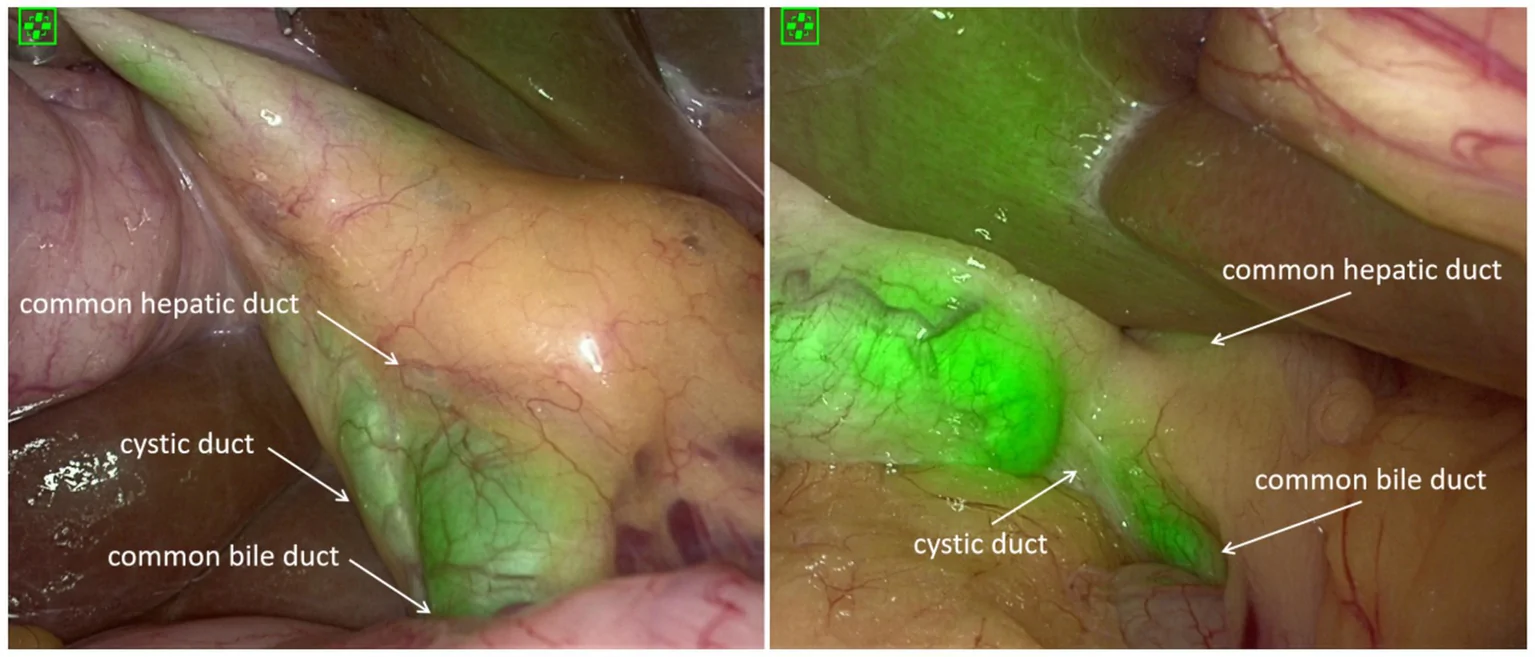
Representative intraoperative image obtained under ICG fluorescence mode before dissection of Calot’s triangle.

**Figure 3:**
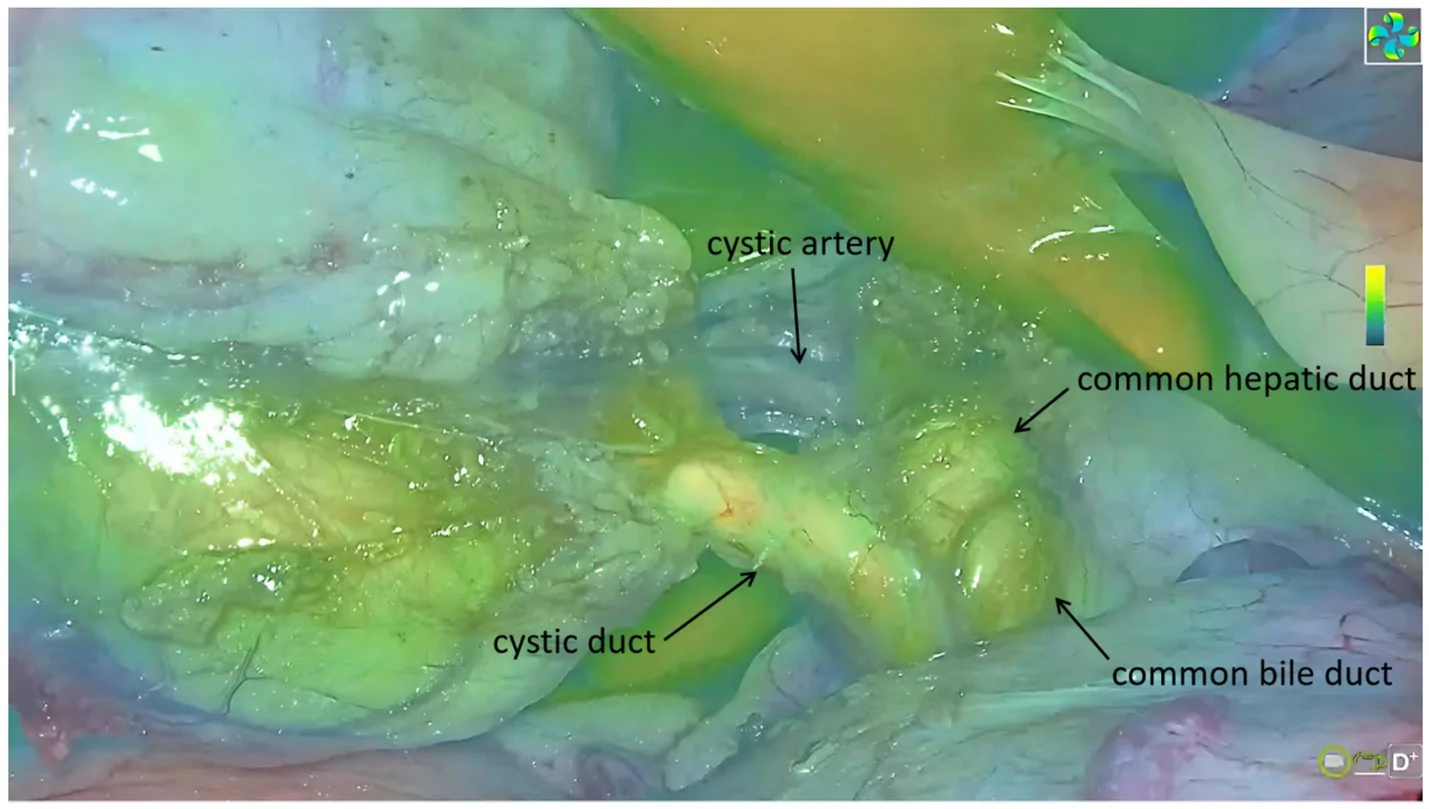
Representative intraoperative image obtained under ICG fluorescence mode after dissection of Calot’s triangle.

Representative intraoperative images obtained under ICG fluorescence mode before and after Calot’s triangle dissection are presented in Figures 2, 3.

Near-infrared fluorescence imaging shows the gallbladder, cystic duct, common bile duct, and common hepatic duct before dissection of Calot’s triangle.

After dissection of Calot’s triangle, fluorescence imaging delineates the cystic duct and its relationship with the common bile duct and common hepatic duct.

### 3.3 Perioperative outcomes

Perioperative outcomes are presented in Table 3. CVS was successfully achieved in all 198 patients. The ICG fluorescence group had a significantly shorter time to achieve the CVS than the conventional white-light group, 12.0 min, interquartile range 9.0–15.5 min, versus 21.0 min, interquartile range 17.0–23.5 min, *p* < 0.001.

**Table 3:** Comparison of perioperative outcomes between groups.

Variable	ICG fluorescence group (*n* = 103)	Conventional white-light group (*n* = 95)	Statistical test	*p* value
Time to achieve CVS (min)	12.0 (9.0–15.5)	21.0 (17.0–23.5)	Mann–Whitney U test	<0.001
Operative time (min)	36.0 (29.0–43.0)	48.0 (41.0–54.0)	Mann–Whitney U test	<0.001
Intraoperative blood loss (mL)	10.0 (5.0–15.0)	10.0 (5.0–15.0)	Mann–Whitney U test	0.329
Drainage duration (d)	0.0 (0.0–0.0)	0.0 (0.0–2.0)	Mann–Whitney U test	<0.001
Postoperative hospital stay (d)	1.0 (1.0–2.0)	1.0 (1.0–2.0)	Mann–Whitney U test	0.785
Conversion to open surgery	0 (0.0)	0 (0.0)	—	—
Drainage placement	18 (17.5)	37 (38.9)	Chi-square test	<0.001

Continuous variables are presented as mean ± SD or median (IQR) according to distribution; categorical variables are presented as *n* (%). CVS: critical view of safety.

Operative time was also significantly shorter in the ICG fluorescence group than in the conventional white-light group, 36.0 min, interquartile range 29.0–43.0 min, versus 48.0 min, interquartile range 41.0–54.0 min, *p* < 0.001.

There was no significant difference in intraoperative blood loss between the two groups. The median intraoperative blood loss was 10.0 mL, interquartile range 5.0–15.0 mL, in both groups, *p* = 0.329. No patient in either group required conversion to open surgery, subtotal cholecystectomy, or another bailout procedure.

Drainage placement was significantly less frequent in the ICG fluorescence group than in the conventional white-light group, 18 of 103 patients, 17.5%, versus 37 of 95 patients, 38.9%, *p* < 0.001. Drainage duration was also shorter in the ICG fluorescence group, 0.0 days, interquartile range 0.0–0.0 days, compared with the conventional white-light group, 0.0 days, interquartile range 0.0–2.0 days, *p* < 0.001.

Postoperative hospital stay did not differ significantly between groups. The median postoperative hospital stay was 1.0 day, interquartile range 1.0–2.0 days, in both groups, *p* = 0.785.

### 3.4 Postoperative complications and safety outcomes

Postoperative complications and safety outcomes are shown in Table 4. No BDI, bile leakage, or reoperation occurred in either group.

**Table 4:** Comparison of postoperative complications and safety outcomes.

Variable	ICG fluorescence group (*n* = 103)	Conventional white-light group (*n* = 95)	Statistical test	*p* value
Bile duct injury	0 (0.0)	0 (0.0)	—	—
Bile leakage	0 (0.0)	0 (0.0)	—	—
Intra-abdominal infection	4 (3.9)	3 (3.2)	Fisher’s exact test	1.000
Incision infection	1 (1.0)	2 (2.1)	Fisher’s exact test	0.608
Reoperation	0 (0.0)	0 (0.0)	—	—
30-day readmission	3 (2.9)	8 (8.4)	Chi-square test	0.091
ICG-related adverse events	0 (0.0)	—	—	—

Data are presented as *n* (%). ICG-related adverse events were applicable only to the ICG fluorescence group.

Intra-abdominal infection occurred in 4 of 103 patients, 3.9%, in the ICG fluorescence group and in 3 of 95 patients, 3.2%, in the conventional white-light group, with no significant difference between groups, *p* = 1.000. Incision infection occurred in 1 patient, 1.0%, in the ICG fluorescence group and in 2 patients, 2.1%, in the conventional white-light group, *p* = 0.608.

Thirty-day readmission occurred in 3 of 103 patients, 2.9%, in the ICG fluorescence group and in 8 of 95 patients, 8.4%, in the conventional white-light group. Although the readmission rate was numerically lower in the ICG fluorescence group, the difference did not reach statistical significance, *p* = 0.091.

No ICG-related adverse events were observed in the ICG fluorescence group.

### 3.5 Multivariable analysis

The results of multivariable analysis are shown in Table 5. After adjustment for age, sex, body mass index, American Society of Anesthesiologists classification, hypertension, diabetes mellitus, previous abdominal surgery, acute cholecystitis, and gallbladder wall thickness, ICG fluorescence navigation remained associated with a shorter time to achieve CVS. The adjusted regression coefficient was −7.91 min (95% confidence interval, −9.00 to −6.81; *p* < 0.001).

**Table 5:** Multivariable analysis of outcomes associated with ICG fluorescence navigation.

Outcome	Model	Comparison	Adjusted covariates	Effect estimate	95% CI	*p* value
Time to achieve CVS (min)	Multivariable linear regression (robust standard errors)	ICG fluorescence group vs. conventional white-light group	age, sex, BMI, ASA, hypertension, diabetes mellitus, previous abdominal surgery, acute cholecystitis, and gallbladder wall thickness	*β* = −7.91	−9.00 to −6.81	<0.001
Operative time (min)	Multivariable linear regression (robust standard errors)			*β* = −12.86	−15.20 to −10.51	<0.001
Postoperative hospital stay (d)	Multivariable linear regression (robust standard errors)			*β* = −0.08	−0.24 to 0.09	0.350
Complete identification of all four biliary structures	Multivariable logistic regression			OR = 9.47	3.96 to 22.62	<0.001
Drainage placement	Multivariable logistic regression			OR = 0.31	0.16 to 0.62	<0.001

Effect estimates compare the ICG fluorescence group with the conventional white-light group. A negative *β* indicates a lower value of the continuous outcome in the ICG group; OR>1 indicates higher odds and OR<1 indicates lower odds in the ICG group.

ICG fluorescence navigation also remained associated with shorter operative time after adjustment, with an adjusted regression coefficient of −12.86 min (95% confidence interval, −15.20 to −10.51; *p* < 0.001).

For postoperative hospital stay, ICG fluorescence navigation was not significantly associated with a reduction after adjustment, with an adjusted regression coefficient of −0.08 days, 95% confidence interval −0.24 to 0.09, *p* = 0.350.

In the logistic regression model, ICG fluorescence navigation was strongly associated with complete identification of all four biliary structures. The adjusted odds ratio was 9.47, 95% confidence interval 3.96 to 22.62, *p* < 0.001.

ICG fluorescence navigation was also associated with a lower likelihood of drainage placement. The adjusted odds ratio was 0.31, 95% confidence interval 0.16 to 0.62, *p* < 0.001.

## 4 Discussion

Among the 198 patients studied, ICG fluorescence was associated with more frequent visualization of all four prespecified biliary structures. At the median, the ICG group reached CVS 9 min earlier and completed the operation 12 min earlier; drains were also placed less often. These differences persisted after adjustment for the measured clinical covariates. Because patients chose whether to undergo ICG-assisted surgery, rather than being assigned at random, the results describe associations and do not establish causation.

The largest between-group difference concerned complete identification of the four biliary structures. Misinterpretation of the cystic duct, common bile duct, or common hepatic duct is a recognized mechanism of BDI (1, 2, 4), especially when inflammation, fibrosis, or adhesions distort Calot’s triangle (5–7). Fluorescence outlines bile-containing structures before and during dissection and may help the surgeon maintain orientation. It does not replace careful dissection or a properly achieved CVS.

Our findings were consistent with the FALCON trial and other comparative studies. In the international FALCON trial, NIR fluorescence enabled earlier identification of extrahepatic biliary anatomy and earlier achievement of CVS than conventional imaging (12). Trials and comparative studies in elective and emergency LC have also reported better visualization of key structures and, in some settings, shorter imaging or operative times (13–16, 20, 21). This evidence makes the differences observed in our cohort plausible, although selection bias, surgeon behavior, and unmeasured factors remain possible explanations.

The four-structure composite was intended to capture the relationship between the cystic duct and adjacent ducts rather than visualization of a single landmark. In the ICG group, the cystic duct and common bile duct were identified in every patient, and identification of the common hepatic duct and cystic duct-common bile duct junction was also more frequent. Seeing the cystic duct in relation to the adjacent ducts may be particularly useful when it is short, wide, tortuous, or abnormally inserted. We did not measure misidentification events or prevention of BDI, so this advantage should be interpreted as improved visualization rather than proof of greater safety.

CVS was achieved a median of 9 min earlier in the ICG group, and the adjusted difference was approximately 7.9 min. A prolonged dissection around Calot’s triangle often reflects greater operative difficulty and may expose the surgeon to less secure planes (6, 7, 22). Earlier visualization of the ducts may help with selection of the dissection plane and confirmation of anatomy before clip application. The quality of CVS itself, however, was not scored independently with a validated system.

Total operative time was also shorter in the ICG group, with a median difference of 12 min that remained significant after adjustment. Published estimates have varied (12–21), probably because patient complexity, surgeon experience, imaging systems, ICG dose and timing, and routine versus selective use differ across studies. In our cohort, the shorter operation was consistent with earlier recognition of the anatomy and faster achievement of CVS. Here, operative efficiency refers only to time to CVS and total operative time; it does not encompass staffing, resource use, cost, or overall procedural quality.

Drain use requires separate interpretation. Surgeons generally base this decision on inflammation, oozing, bile contamination, difficulty of dissection, uncertainty about anatomy, and concern about postoperative collections. Clearer anatomy may have influenced confidence at the end of the procedure, but drain placement was not protocolized. This endpoint therefore reflects clinical judgment as well as the operative course and should not be treated as a direct measure of efficiency or a causal benefit of ICG.

Major adverse outcomes were uncommon: neither group had BDI, bile leakage, conversion to open surgery, or reoperation, and no ICG-related adverse event was recorded. The cohort is too small to determine whether ICG prevents BDI. A 2025 meta-analysis of randomized trials found better common bile duct identification but no statistically demonstrated reduction in BDI or other rare patient-centered outcomes (17). A 2026 review reached a similar conclusion despite higher common bile duct and common hepatic duct identification rates with NIR fluorescence (23). Our safety findings should therefore be limited to short-term feasibility and process outcomes.

ICG dosing remains unsettled. We used a fixed 1-mg dose, reconstituted to 2.5 mg/mL and given 30 min before surgery. Recent trials show that image quality depends on both dose and timing. DOTIG compared several dose-interval combinations (24); Lin et al. compared intraoperative with preoperative administration using a very low intraoperative dose (25); Huang et al. studied low-dose administration within 30 min of LC (26); and Broderick et al. examined intraoperative microdosing (27). Ladd et al. found that a low-dose regimen improved the bile duct-to-liver contrast by reducing hepatic background fluorescence (28), while a 2024 double-blind trial found dose-related differences in duct detection (29). The 1-mg regimen used here was therefore a practical low-dose protocol, not a claim of optimal dosing. Its performance may vary with body mass index, hepatic function, inflammation, the injection-to-imaging interval, and the sensitivity of the imaging platform.

Given the low incidence of BDI, the present cohort was informative for process outcomes but not for injury rates. Anatomical visualization and time to CVS remain intermediate outcomes and cannot be assumed to translate into fewer injuries, lower morbidity, or better long-term recovery.

Strengths of the analysis include the consecutive clinical cohort, a primary endpoint that required identification of four biliary structures rather than one, and parallel assessment of anatomical and perioperative outcomes. The adjusted models included age, sex, body mass index, American Society of Anesthesiologists classification, hypertension, diabetes mellitus, previous abdominal surgery, acute cholecystitis, and gallbladder wall thickness.

The main limitation is the non-random allocation. Patients selected the surgical approach after counseling, so the two groups may have differed in ways that were not captured in the dataset. Multivariable adjustment addresses measured covariates but cannot remove self-selection bias or residual confounding. The observed differences should therefore not be interpreted as causal effects of ICG.

The primary endpoint also depended on clinical judgment. The operating surgeon assessed the anatomy during the procedure, and one senior physician reviewed the video afterward. The review was neither blinded nor performed by several independent assessors, and inter-rater agreement was not measured. Knowledge of the imaging mode may therefore have influenced classification. A prospective study should use prespecified video criteria, blinded reviewers, and formal assessment of agreement.

Drain placement was not protocolized and remained dependent on the surgeon’s assessment of the operation. The two measures labeled operative efficiency were limited to time to CVS and total operative time; resource use, cost, and a validated composite measure were not evaluated. Three fluorescence platforms were used, and differences in illumination, detector sensitivity, image processing, or display were not quantified.

The cohort was not large enough to evaluate rare outcomes such as BDI, bile leakage, conversion, or reoperation. No subtotal cholecystectomy or other bailout procedure was required, so the findings may not apply to the most difficult cases. We also did not measure fluorescence intensity, bile duct-to-liver contrast, operative difficulty with a validated scale, cost-effectiveness, or long-term outcomes. As a single-center study using local practice patterns and three imaging systems, its generalizability is uncertain.

Further evaluation should be prospective and multicenter, with controlled allocation, standardized ICG administration and system settings, validated grading of operative difficulty, blinded video review, and predefined criteria for drain placement. Large registries or adequately powered trials will be needed to study BDI. Recent dose-comparison trials and evidence syntheses also show why administration and imaging protocols should be standardized before the findings are generalized broadly (23, 28, 29).

## 5 Conclusion

In this preference-allocated, single-center cohort, ICG fluorescence use was associated with more frequent identification of the prespecified biliary structures, earlier achievement of CVS, shorter operative time, and less frequent drain placement. No ICG-related adverse event was recorded. The observational design, judgment-dependent outcomes, and limited sample size preclude conclusions about causality or BDI prevention. ICG fluorescence may be used as an adjunct to careful dissection and CVS; prospective multicenter studies with standardized protocols are needed.

## Data Availability

The original contributions presented in the study are included in the article/supplementary material, further inquiries can be directed to the corresponding authors.
